# Quality of Life After Bariatric Surgery

**DOI:** 10.1007/s11695-015-1601-2

**Published:** 2015-02-13

**Authors:** Piotr Major, Maciej Matłok, Michał Pędziwiatr, Marcin Migaczewski, Piotr Budzyński, Maciej Stanek, Michał Kisielewski, Michał Natkaniec, Andrzej Budzyński

**Affiliations:** 2nd Department of Surgery UJCM, Jagiellonian University, ul. Kopernika 21, 31-501 Kraków, Poland

**Keywords:** Obesity, Quality of life, Sleeve resection, Gastric bypass, Bariatric surgery, SF-36

## Abstract

**Introduction and Purpose:**

Morbid obesity together with obesity-related diseases has a negative impact on the quality of life. The aim of the study was to assess the quality of life amongst patients with morbid obesity as well as the impact of bariatric treatment on body weight and obesity-related diseases in addition to conducting an analysis of changes in the quality of life after surgical treatments, in the context of the surgical procedure type and degree of body weight loss.

**Material and Methods:**

Sixty-five patients were treated for morbid obesity. The sample group consisted of 34 patients treated with laparoscopic sleeve gastrectomy (LSG) and 31 persons qualified for laparoscopic Roux-en-Y gastric bypass (LRYGB). The average body weight before the procedure was 146.2 kg. In the sample group, 89 % of persons qualified for the surgical treatments were diagnosed with hypertension and 52 % persons that were operated on were diagnosed with diabetes type 2 before the surgical procedure. Before commencement of the surgical treatment, the quality of life was assessed, which in both groups qualified for given types of bariatric procedures was considerably low.

**Results and Conclusions:**

Percentage excessive weight loss (%EWL) was 58.8 %. No significant differences in body weight loss were noted between the two types of procedures. Improvement was observed in the treatment of obesity-related diseases. Also, the quality of life was enhanced significantly. No differences were noted in terms of the quality of life improvement between particular types of surgical procedures. No significant differences were observed during the analysis of body weight loss impact on the quality of life improvement.

## Introduction

In the developed and developing countries alike, the obesity rate rapidly increases, already reaching an epidemic level [1]. In Poland, about 300 thousand people have a body mass index (BMI) above 40 kg/m^2^, while in over 1.5 million, the BMI exceeds 35 kg/m [2]. The continuous increase in a number of obese people is accompanied by an increase in a number of obesity-related diseases, including type 2 diabetes, hypertension, lipid disorders, or ischaemia disease [3].

In 1946, the World Health Organisation in its constitution defined health as a state of complete physical, mental, and social well-being and not merely the absence of disease or infirmity. Therefore, the modern concept of health also covers the quality of life, and a sense of health is one of its basic determinants. According to many authors, a sense of health is, actually, a fundamental condition determining a good quality of life. Amongst numerous complications related to obesity and overweight, apart from somatic diseases affecting function of individual organs and systems, their negative effect on the quality of life is increasingly often mentioned.

A study was planned to analyse issues concerning effects of laparoscopic bariatric surgeries in patients operated for super obesity, with particular focus on the effect of surgical treatment on the quality of life of treated people.

## Aim of the Paper

The aim of this paper was to analyze the bariatric surgery effect on the quality of life, considering the type of surgery and the weight loss rate.

## Methods

A prospective study was conducted in a group of patients with super obesity, treated at the 2nd Department of Surgery UJCM, Jagiellonian University. Patients with super obesity enrolled in the study met criteria qualifying for a bariatric surgery, i.e.:

- BMI exceeding 40 kg/m^2^
- BMI exceeding 35 kg/m^2^, when diagnosed with obesity-related diseases, including type 2 diabetes, hypertension, lipid disorders, and obstructive sleep apnea
- age 18–65 years.

The study was approved by the Bioethics Committee of the Jagiellonian University. All patients included in the study group gave their written consent for participation in the study after being thoroughly informed on assumptions and aims of the study. The patients qualified for surgery for super obesity underwent an initial screening. The screening included a detailed medical interview, physical examination, anthropometric measurements, and an initial quality of life assessment. The body mass was determined with a specialist body composition analyser, Tanita BC-420S MA. The patients were qualified for one of two of surgical methods. Some patients underwent laparoscopic sleeve gastrectomy (LSG), and the remaining part had laparoscopic Roux-en-Y gastric bypass (LRYGB). Decision about surgery type was taken together with the patients after previous presentation of the possible methods of treatment, their efficiency, and potential complication. Patients preferences were the main factor in the selection of the surgery type. After 1 year from the surgical treatment, the patients were invited to a follow-up visit, during which anthropometric parameters and the quality of life were reassessed. The obtained results were analysed statistically.

### Quality of Life Assessment

The quality of life in patients treated surgically for morbid obesity at the 2nd Department of Surgery UJCM, Jagiellonian University, was assessed with licenced, standardised and developed for medical purposes forms, SF-36 (*Short Form Health Survey*) and MA-QoLQII (*Moorehead-Ardelt Quality of Life Questionnaire II*).

### Statistical Analysis

The statistical analysis was conducted with the *STATISTICA 10 PL* package. In all analyses, all effects for which the probability value, *p*, was below the assumed level of significance, *α* = 0.05 (*p* < 0.05), were identified as significant.

## Material

The study involved 65 patients who underwent LSG or LRYGB for morbid obesity at the 2nd Department of Surgery UJCM, Jagiellonian University in a period from October 2011 to May 2013. The group included 39 women (of the average age 44.4 years) and 26 men (of the average age 41.1 years). The average age in the whole group was 42.75 years. LSG was performed in 34 people, and LRYGB was performed in 31 patients.

The average body mass before the surgery was 146.2 kg (ranging from 112 to 196 kg), and the average BMI value before the surgery was 50.44 kg/m^2^ (ranging from 39.7 to 71.13 kg/m^2^). The average body mass index (BMI) values in the group of patients qualified for LSG and for LRYGB were 49.98 and 50.72 kg/m^2^, respectively. No statistically significant difference was observed between the groups (*p* = 0.7).

In the studied group, hypertension was diagnosed in 58 (89 %), and type 2 diabetes was diagnosed in 34 (52 %) people qualified for the surgery. Of 34 patients treated for diabetes, 29 (85.3 %) required insulin therapy. Patients with super obesity diagnosed with concurrent type 2 diabetes and hypertension were diagnosed with metabolic syndrome. This group included 13 (22 %) patients in total. Various other complications of super obesity were observed in participants. In the group of 65 patients, 44 (67 %) were diagnosed with lipid disorders. Obstructive sleep apnea occurred in 11 (16.9 %) of the patients. All patients qualified for surgery were diagnosed with non-alcoholic hepatic steatosis in the ultrasound scan, and in five, (7.69 %) cholelithiasis was observed in the gallbladder, previously without symptoms. Myocardial ischaemia affected seven (10.7 %) of the patients, of which four (6.1 %) had a history of cardiac infarction.

## Results

### Pre-surgery Quality of Life Assessment

On a basis of answers to questions in the SF-36 questionnaire provided before the planned surgery, values identifying the quality of life in terms of physical and mental health were calculated. The average value of the global quality of life index was 85.2 for the whole studied group. In the group of patients qualified for LSG, the global quality of life index was statistically significantly lower (69.9) than in the group qualified for LRYGB (102.06) (*p* = 0.00). Similarly, a statistically significant difference was observed for parameters assessing the quality of life in terms of physical function, vitality, and limitations in social roles resulting from emotional problems, as well as in terms of a sense of mental health.

On a basis of answers given by patients to questions in the Moorhead-Ardelt Quality of Life Questionnaire II, concerning five basic aspects of the quality of life, i.e. general self-esteem, physical activity, social contacts, work, sexual activity, and focus on eating behaviour, the global quality of life was also assessed, and then qualified into one of the five categories. In one patient (1.6 %), the quality of life was assessed as very bad, in 14 (21.5 %) patients, as bad, and in 38 (58.4 %), as an average. The quality of life assessed as good and as very good was described in nine (13.8 %) and in three (4.7 %) patients, respectively. No statistically significant difference (*p* = 0.34) was noted between patients treated with LSG and with LRYGB.

### Surgical Treatment Results

Following the surgical treatment, a significant body mass reduction was achieved in all patients. The average body mass index, BMI, measured in the studied group during the follow-up visit 12 months post surgery was 33.4 kg/m^2^ (ranging from 25.69 to 46.81 kg/m^2^). The average percentage excessive weight loss (%EWL) was 58.8 %, and the average body mass index loss (%EBMIL) was 68.85 %. No statistical difference was observed between groups of patients qualified for individual surgery types for the post-surgery body mass reduction.

During the follow-up visit, the significant reduction in severity of comorbidities was also observed. Type 2 diabetes and hypertension were resolved in 26 (76.5 %) of 34 people, and 42 (72.4 %) of 58 people, respectively. In remaining patients, the significant reduction of doses and numbers of medications for treatment of comorbidities were observed. Similarly as for body mass reduction, no significant differences were noted for reversal of chronic diseases related to obesity.

### Post-Surgery Quality of Life Assessment

**Fig. 1:**
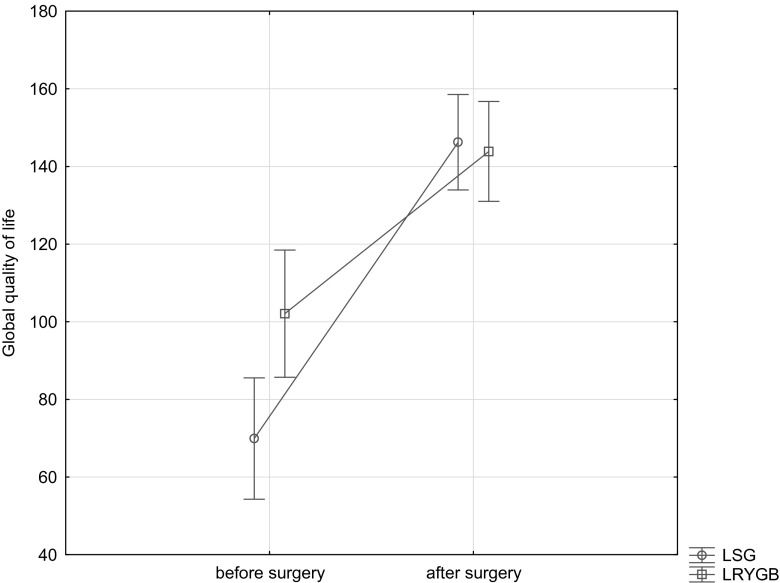
The global quality of life in the group of patients post surgery, depending on the surgery type

For all quality of life parameters assessed with the SF-36 form, statistically significant change was noted in time. The average value of the global quality of life index was 145.1 and was statistically significant versus the initial value. In the group of patients qualified for LSG and for LRYGB, the average global quality of life index was 146.2 and 143.8, respectively (*p* = 0.00). The initial difference noted in pre-surgery measurement disappears in the final measurement. This results from faster increase on the quality of life in the group of post-LSG patients (Fig. 1).

A statistically significant difference between relevant surgery types was observed solely for a parameter evaluating limitations in social roles resulting from emotional problems (*p* = 0.00). For other parameters, no statistically significant differences were observed for final values of individual parameters between the two types of surgeries (Table 1).

**Table 1:** Summary of results for the SF-36 questionnaire obtained post surgery

Quality of life		LSG	LRYGB	*p*
Physical function	Before surgery	34.1	39.5	0.04
	After surgery	55	51.9	0.06
Role physical	Before surgery	32.8	38.9	0.13
	After surgery	55	49.6	0.21
Body pain	Before surgery	46.7	43.3	0.28
	After surgery	57.3	54.5	0.85
General health	Before surgery	37.6	39.5	0.32
	After surgery	53.7	50.6	0.48
Vitality	Before surgery	42.4	48.7	0.00
	After surgery	55.9	55.1	0.97
Social function	Before surgery	42.59	43.6	0.36
	After surgery	51.73	51.67	0.95
Role emotional	Before surgery	31.28	41.79	0.03
	After surgery	52.5	49.4	0.00
Menthal health	Before surgery	38.3	45.63	0.00
	After surgery	49.5	49.6	0.91
Global quality of life	Before surgery	69.9	102.06	0.00
	After surgery	146.2	143.8	0.61
Global quality of life – physical helth	Before surgery	39.13	39.51	0.68
	After surgery	56.9	52.61	0.22
Global quality of life – mental helth	Before surgery	38.7	46.88	0.00
	After surgery	50.17	50.41	0.26

**Fig. 2:**
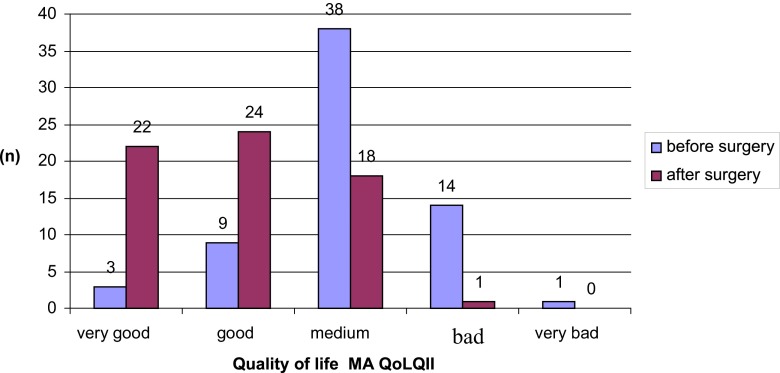
An analysis of changes in the quality of life pre- and post-surgery, as determined with the Moorhead-Ardelt Quality of Life Questionnaire II

The quality of life post surgery was also determined with the Moorhead-Ardelt Quality of Life Questionnaire II. No patient was assessed as very bad, in one (1.51 %) patient, the quality of life was assessed as bad, and in 18 (27.89 %), as an average. The quality of life was assessed as good and very good in 24 (36.8 %) and in 22 (33.8 %) patients, respectively (Fig. 2).

### Rate of Body Mass Reduction and Quality of Life

**Fig. 3:**
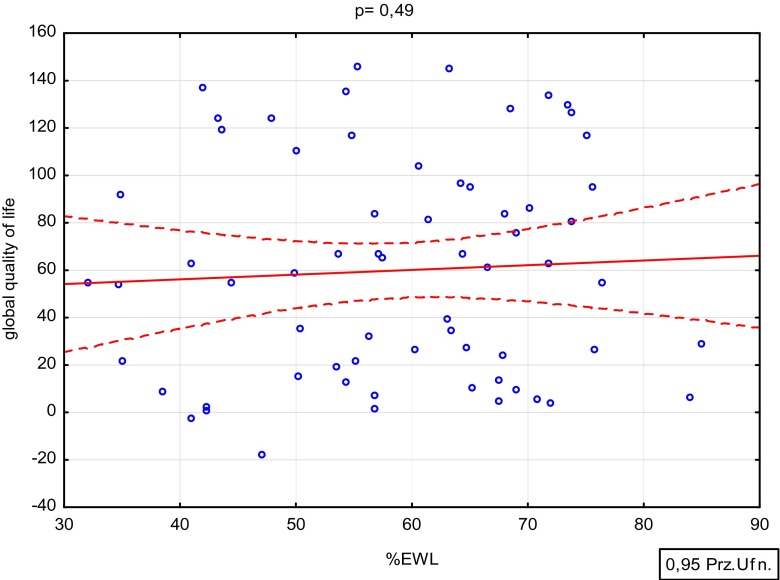
%EWL effect on changes in the quality of life post surgery

To answer the question whether a rate of body mass loss influences a degree of the quality of life improvement, the obtained index for assessment of bariatric treatment effectiveness (%EWL) was correlated with average changes in the global quality of life. The non-parametric Spearman’s correlation was used for a statistical analysis. No significant correlation was found for analysed variables (Fig. 3).

## Discussion

A continuous increase in a number of people with excessive body mass observed in the last decade has made the obesity and its related diseases one of the main health problems in the contemporary world. The epidemiological data from Poland do not deviate from global tendencies; also, in our country, the number of people suffering with overweight and obesity increases, including the number of patients with an extreme form of obesity, called morbid obesity [2].

Increasingly often, besides health deterioration resulting from obesity, psychological, emotional, or social aspects related to it are noticed, which together have a negative effect on the quality of life. The importance of the quality of life in the therapeutic process is confirmed by numerous studies evaluating the effect of chronic diseases, including super obesity, on well-being and satisfaction with life in treated patients.

The fact of the negative effect of obesity and its related diseases on the quality of life is commonly known [4]. Numerous clinical studies and publications showed clearly that the quality of life of obese people is worse than of people with correct body weight. This concerns not only the quality of life related to physical function, for example, intolerance of physical exercise, problems with moving, or pain, but also the quality of life related to psychical aspects, including lack of acceptance of themselves, increased stress level, reduced self-esteem and mood, or depressive states [5]. A continuous increase in the number of obese people is, therefore, tantamount to a continuous increase in the group of people with reduced quality of life, in its physical and mental dimensions.

Current expectations of patients suffering with super obesity towards medical care include actions not only improving their health but also leading to their psychical and emotional well-being [6]. An effective method for treatment of patients with morbid obesity, resulting in a permanent weight loss, is surgical treatment. BMI reduction post surgery is also accompanied by advantageous metabolic changes, which in some patients result in observed reduced severity or complete reversal of diseases caused by obesity. Thus, the reduced body mass together with a general health improvement lead to improvement on the quality of life of treated patients.

Therefore, improvement on the quality of life, besides treatment of obesity and diseases accompanying it, has become one of the main aims of bariatric treatment.

During the follow-up visit that took place 1 year after the surgery, the average body mass of studied patients was 97.1 kg, and the average BMI was 33.4 kg/m^2^, and this means an average body mass reduction of 51.55 kg (17.48 kg/m^2^). The obtained results are statistically significant (*p* = 0.00). What is interesting, no statistical differences were observed between individual types of surgeries (*p* = 0.52). These results do not differ from data that can be found in the literature. For example, in the Coupaye report, the body mass loss 1 year post sleeve gastrectomy and post mini-gastric bypass was 35.9 and 38.6 kg, respectively. The final results in both groups were similar [7]. Whereas in the Swedish study, Targerson and Siöström showed an average body weight reduction of 28 kg. The average percentage excessive weight loss (%EWL) post surgery was 58.8 %. The results achieved after the two types of surgery were similar and did not differ significantly (*p* = 0.10). Assuming that loss of 50 % of the excessive body mass is considered a satisfactory treatment effect, the obtained results seem to be fully satisfying and allow to announce a therapeutic success. For comparison, a similar percentage excessive weight loss in the study reported by Lakdawal was 50.8 %. The authors also did not note significant differences between LSG and LRYGB in their study [8]. Likewise, in the Boza study, similar effects were achieved for the two types of surgery, and no significant differences were observed for %EWL between these procedures [9]. What is interesting, when compared to the publications quoted above, the group of patients being the subject of our study achieved the highest reduction in body mass following the applied treatment. Observed differences may result from a different initial body weight which in our group was the highest.

With the loss of body mass, 1 year post surgery, an improvement in treatment of all diseases caused by obesity was observed in our group of patients. The improvement was seen in patients treated with the LSG and the LRYGB procedures alike. Also in studies of Paluszkiewicz and Boza, no significant difference was seen in reversal of chronic diseases according to a selected type of surgical procedure [10].

A currently applicable definition of health proposed by WHO, defining health as a state of complete physical, mental, and social well-being and not merely the absence of disease or infirmity, formed a basis for a term for the quality of life in general and health-related quality of life (HRQOL) [11]. Available tools for assessment of the quality of life include questionnaires for a general assessment and those used to evaluate the quality of life in specific conditions, including super obesity. In our study to assess the quality of life of patients surgically treated for super obesity, the SF-36 questionnaire (*Short Form Health Survey*) was used. This questionnaire is the most commonly used tool in clinical studies assessing the health-related quality of life. It contains only 36 simple questions, and its completion is not time-consuming. Its Polish version was developed, contrary to other questionnaires. Furthermore, pilot studies were conducted, which results were used to develop standard tables validated for the population in our country. This questionnaire is one of the most commonly used for evaluation of the quality of life of patients with somatic disorders. To evaluate the quality of life in a specific group of patients, for people suffering from super obesity, the MA QoLQII questionnaire developed on a basis of the BAROS questionnaire especially for bariatric surgery needs was also used. Similar methods for assessment of the quality of life were also used by other authors, such as D’Hondt or Al Harakeh. Following analysis of available publications, it seems that methods selected in this paper for assessment of the quality of life of patients with super obesity qualified for surgery were selected correctly.

In analysing initial results obtained in the assessment with the SF-36 questionnaire conducted before surgical treatment at the 2nd Department of Surgery UJCM, Jagiellonian University, a significantly reduced quality of life was found in the whole group. The quality of life in the group qualified for LSG was lower. The reason for observed differences was not found, and the groups did not differ significantly in their demographic structure, initial body mass, and presence of diseases related to obesity. In the whole studied group, the physical dimension of the quality of life expressed as points was 39.9. Assuming that 50 points are considered to be an average norm, the obtained results indicate a significant reduction on the quality of life in terms of physical health in treated patients. No statistically significant differences were seen between groups qualified for specific surgical procedures (*p* = 0.68). However, a significant difference between groups was recorded for initial scores determining the quality of life in its psychical dimension (*p* = 0.0). In the group of patients qualified for LSG and for LRYGB, that value was 38.7 and 46.88, respectively (*p* = 0.00). Therefore, similarly as for the quality of life index, also, the psychical dimension of the quality of life was significantly lower in the group of patients treated with the LSG procedure versus others. The average value for the whole studied group was 42.61 points, significantly differing from the accepted standard, and this signifies a deteriorated quality of life in its psychical dimension in all treated people. In Sarwer’s study, in analysing the effect of the LRYGB procedure on the quality of life of operated patients, the SF-36 questionnaire was also used. The quality of life index value was only 52.3. The quality of life index in its physical and psychical dimensions was estimated to be 48.7 and 62 points, respectively. Thus, these values were lower than in the group studied by us [12]. Martina de Zwaan in her paper also presented results based on the SF-36 questionnaire determining the quality of life in its physical and psychical aspects as 30.6 and 48.9 points, respectively. Also in this case, the quality of life was lower than in the group studied by us. However, the study did not include the quality of life index.

Similar results, indicating significant reduction on the quality of life of patients with super obesity, were obtained by analysing information obtained in the Moorehead-Ardelt Quality of Life Questionnaire II. In the studied group before the surgery, the quality of life was lower in over 80 % of people, of which 23.1 % described it as bad or very bad. The obtained data corresponds to study results of other authors [13].

One year post surgical treatment, a significant improvement on the quality of life was noted. When assessing results in the SF-36 questionnaire, the observed changes concerned the global quality of life index, physical and psychical dimensions, as well as other determinants of the quality of life. For all studied indices, changes in their values in time were statistically significant (*p* = 0.00). In the whole studied group, the quality of life in terms of physical functioning 1 year post surgery was 53.5 points. This value visibly exceeds the standard for the Polish population, and this may seem strange, as hardly any of the patients achieved a correct, optimal body weight following bariatric treatment. Despite still being overweight or even obese, the quality of life post surgery was better than the hypothetical quality of life of an overweight person not qualifying for surgical treatment. Some patients post surgery still required treatment for diseases accompanying obesity, but still, their quality of life was higher than average. Analogous observations were made when analysing individual determinants for the quality of life. Patients treated with the LSG and LRYGB procedures achieved a similar final result (*p* = 0.06). Despite a significantly lower initial value of the studied parameter in the group qualified for sleeve gastrectomy, the quality of life in both groups reached a similar level after the end of the treatment. This means that in the group of patients post sleeve gastrectomy, the improvement was more pronounced and faster. Summarising results obtained on a basis of the SF-36 questionnaire, the quality of life improved versus values determined before surgical treatment. The quality of life index rose from 85.2 to 145.1 points (*p* = 0.00). Patients post both surgeries achieved a similar end result (*p* = 0.61). What is interesting, the group of patients treated with the LSG procedure, initially characterised by significantly worse quality of life, achieved a similar effect with the surgery as the group treated with the LRYGB procedure due to a faster and higher increase. The analogous relationship was observed when physical aspects of the quality of life were analysed. The obtained results increased significantly from 42.61 to 50.2 points, thus exceeding the standard (*p* = 0.00). The quality of life level related to mental health was the same in both groups post surgery (*p* = 0.26), and the difference existing before the procedure disappeared due to a higher and faster improvement rate in the group of people operated with the LSG procedure. Also, in its physical aspect, the quality of life significantly improved post surgery (*p* = 0.00). No statistically significant differences on the quality of life improvement were observed for the physical dimension between the two types of surgeries (*p* = 0.22). It also seems interesting that in the studied group, no extremely good quality of life was noted. This means that the level achieved by operated people, although relatively higher than the standard, does not reach maximum values.

In similar studies, de Zwaan presented results concerning the quality of life assessed on a basis of the SF-36 questionnaire in the group of patients operated only with the LRYGB method. Similarly to our study, the author observed a significant improvement in all studied parameters. In most cases, their values for the studied population post surgery exceeded the standard [14]. Similar results were also presented by Sarwer. What is interesting, a significant improvement for all parameters specified in the questionnaire was noted already after 20 weeks from the surgical treatment, and with time, changes progressed further, but to a much smaller extent. This was a period in which the body mass reduction did not reach its final value and diseases accompanying obesity probably were not reversed. This supports our hypothesis that the very fact of having a surgery resulted in a significant improvement on the quality of life. The results obtained in our study are therefore consistent with reports of other authors.

When analysing results obtained with the MA QoLQII questionnaire post surgery, it was found that the quality of life assessed with this tool also was improved after surgical treatment. In as many as 70.6 % of 65 patients, the quality of life post bariatric treatment was assessed as very good or good. In the group of remaining people, in the significant majority, the quality of life was assessed as average, that is, corresponding to the general standard. The obtained results did not differ, depending on the type of surgery. Al Harakeh presented similar results concerning improvement on the quality of life post bariatric surgery, as estimated on a basis of the BAROS questionnaire [15]. Also Bobowicz, analysing data from the Polish centre, observed a significant improvement on the quality of life post surgery. Thus, the quoted studies present data consistent with results reported in this paper.

When analysing further results, it was found that also a degree of the body mass reduction post surgery does not influence the degree of quality of life improvement. Both people with low and high values for parameters evaluating the bariatric treatment effectiveness (%EWL, %EBMIL) reached similar improvement on the quality of life. These observations, despite lack of extensive literature on this subject, seem to be the most interesting result of the conducted study. There are opinions in the literature indicating significant differences in treatment results for such diseases as type 2 diabetes, hypertension, or lipid disorders depending on the body mass reduction rate and type of the surgery. However, the results of the conducted study indicate a completely different effect of the bariatric surgeries on the quality of life. It seems that the very fact of bariatric surgery is more important for improvement on the quality of life than the body mass reduction rate. Therefore, following the conducted study, we are of the opinion that there is no relationship between the degree of body mass reduction and the level of improvement on the quality of life. Also, in the Sarwer study, no significant differences were observed for improvement on the quality of life in relation to the degree of body mass reduction [12]. However, data in the literature confirming our observation is scarce. Therefore, it seems necessary to conduct more extensive studies in that area.

In conclusion, it should be emphasised again that for people with super obesity, the very fact of the surgical treatment results in a significant improvement in the quality of their life, regardless of how much it has been deteriorated by the disease. What is interesting and, at the same time, difficult to explain is the fact that the quality of life of patients post surgery reaches the level exceeding the standard value for our population. It should also be emphasised again that the type of conducted surgery seems to be of no importance here. Additionally, it also is surprising that the extent of body weight loss achieved by a patient following the surgery is not proportionally reflected in improvement of the quality of life. The fact that the quality of life post bariatric treatment is so spectacularly improved is a strong argument supporting qualification of patients with super obesity for surgical treatment, despite a high level of difficulty of the procedure and related potential risks. Nonetheless, long-term observation of the quality of life after bariatric surgery seems to be necessary.
